# La préservation du capital vasculaire au CHU Ibn Sina de Rabat-Maroc: rôle de l’infirmier

**Published:** 2012-03-24

**Authors:** Tarik Bouattar, Aicha Bezzaz, Khalil Abdellaoui, Fatima Ezzahra Lamchahab, Loubna Benamar, Fatima Ezaitouni, Badredine Hassam, Rabia Bayahia, Naima Ouzeddoun

**Affiliations:** 1 Service de Néphrologie-Dialyse–Transplantation Rénale, CHU Ibn Sina-Rabat, Maroc, Faculté de Médecine et de pharmacie, Université Mohamed V, Rabat, Maroc; 2 Service de Dermatologie Vénérologie, CHU Ibn Sina Rabat, Maroc, Faculté de Médecine et de pharmacie, Université Mohamed V, Rabat, Maroc

**Keywords:** Infirmier, information, maladie rénale, préservation, veine, cathéter

## Abstract

**Introduction:**

Tout patient ayant une maladie chronique nécessitant des prélèvements sanguins répétés ou des traitements par voie veineuse ou susceptible d’évoluer vers l’insuffisance rénale chronique, doit bénéficier d’une stratégie de préservation de son réseau veineux. Le but de ce travail etait de déterminer le rôle de l’infirmier dans la protection du réseau veineux superficiel.

**Méthodes:**

Etude transversale réalisée au mois d’Avril 2010 à l’hôpital Ibn Sina de Rabat ayant intéressée les infirmiers exerçant dans différents services prenant en charge des patients ayant des pathologies rénales chroniques ou une maladie générale susceptible de se compliquer d’atteinte rénale.

**Résultats:**

Parmi les 80 infirmiers sollicités, 66 ont complété le questionnaire avec un âge moyen de 42,7 ±11ans et un sex-ratio à 0,7. L’ancienneté moyenne en soins était de 227,7 ± 116,7 mois. 37 % des infirmiers n’étaient pas informés sur la préservation du capital veineux. Le membre supérieur droit était ponctionné dans 92,3% des cas. Le site de ponction le plus utilisé était le dos de la main dans 84,6 % des cas. La dilatation des veines par le réchauffement était notée dans 63,3% des cas, par passage d’alcool dans 54,5% des cas et l’emploi du garrot était retrouvé dans 95,5% des cas. Les venojects étaient de 18 gauge dans 18,2 % des cas et de 21 gauge dans 81,8% des cas. Les intranules étaient de 18 gauge dans 78,8% des cas et de 20 gauge dans 21,2 % des cas. Durant les six derniers mois avant l’étude, les complications au niveau du site de ponction étaient notées dans 41 % des cas.

**Conclusion:**

L’information et l’éducation du personnel paramédical sont obligatoires ainsi que l’utilisation des cathéters veineux de petit calibre afin d’assurer cette protection.

## Introduction

Le nombre de patients nécessitent le recours à hémodialyse chronique est approximativement de 300000 aux Etats-Unis [1]. Au Maroc, 4845 patients étaient en hémodialyse intermittente en 2006 [2], les estimations actuelles sont à 10000. Le coût de l’hémodialyse affecte de manière significative les coûts de santé et l’économie nationale. L’amélioration de la stratégie de création des accès vasculaires aurait probablement comme conséquence une augmentation de leur viabilité fonctionnelle, et une réduction de la morbidité et des coûts de santé [3]. Les recommandations de la National Kidney Foundation Kidney Disease Outcomes Quality Initiative (NKF-K/DOQI) préconisent qu’au moins la moitié des patients en insuffisance rénale chronique terminale (IRCT) qui nécessitent une hémodialyse chronique devrait avoir une fistule artérioveineuse (FAV) [4]. La multiplication des ponctions ou des perfusions veineuses conduit à la raréfaction du réseau veineux [5]. La protection du capital veineux va permettre de réaliser ultérieurement un accès vasculaire facilement utilisable afin de mettre en œuvre les différentes techniques d’hémodialyse [6]. Le but de notre travail était de déterminer le rôle de l’infirmier dans la protection du réseau veineux superficiel et d’expliquer les différentes mesures de préservation du réseau veineux superficiel et profond.

## Méthodes

Il s’agit d’une étude transversale réalisée au mois d’Avril 2010 à l’hôpital Ibn Sina de Rabat ayant intéressée les infirmiers exerçant dans différents services médicochirurgicaux prenant en charge des patients ayant des pathologies rénales chroniques ou une maladie générale susceptible de se compliquer d’atteinte rénale à long terme.

L’enquête a été effectuée en se basant sur une fiche d’exploitation d’administration directe et en absence de l’enquêteur. La première partie du questionnaire concernait l’identification des infirmiers ainsi que leur activité. La deuxième partie concernait le siège et le site de ponction, le calibre du matériel et les mesures utilisées pour la dilatation des veines. La troisième partie concernait la fréquence des différentes complications notées au cours des six derniers mois avant l’étude secondaires aux prélèvements et aux perfusions veineuses.

Au plan statistique, les données ont été saisies et analysées à l’aide du logiciel SPSS 13.0. Les variables quantitatives ont été exprimées en moyenne et en écart-type. Les variables qualitatives ont été exprimées en effectif et pourcentage.

## Résultats

Parmi les 80 infirmiers sollicités, 66 ont complété le questionnaire, soit un taux de réponse de 82.5%. Leur âge moyen était de 42,7 ±11ans avec des extrêmes allant de 20 à 58 ans et un sex-ratio à 0,7. L’ancienneté moyenne en soins était de 227,7 ± 116,7 mois (7 à 422). 37% des infirmiers n’étaient pas informés sur la préservation du capital veineux.

Ces infirmiers exerçaient principalement dans les services de néphrologie, cardiologie, endocrinologie, médecine interne, réanimation médicale, urologie et de chirurgie vasculaire.

Le membre supérieur droit était ponctionné dans 92,3% des cas et le membre supérieur gauche dans 81,5% des cas. Le site de ponction le plus utilisé était le dos de la main dans 84,6 % des cas, suivi du pli du coude dans 76,9% des cas et enfin le poignet dans 61,3% des cas.

La dilatation des veines par le réchauffement était notée dans 63,3% des cas, par passage d’alcool dans 54,5% des cas et l’emploi du garrot était retrouvé dans 95,5% des cas.

Les venojects utilisés pour les prélèvements était de 18 gauge dans 18,2 % des cas et de 21 gauge dans 81,8% des cas. Les intranules employées étaient de 18 gauge dans 78,8% des cas et de 20 gauge dans 21,2 % des cas. 66,7% des services utilisaient les épicrâniennes qui étaient de 18 gauge dans tous les cas.

Durant les six derniers mois avant l’étude, les complications au niveau du site de ponction étaient notées dans 41 % des cas: hématome dans 70,4% des cas, lymphangite chez 44,4% des patients et un état de choc anaphylactique ayant bien évolué chez un seul patient.

## Discussion

Dans notre travail, seulement un tiers des infirmiers était informé sur la préservation du capital veineux ce qui concorde avec les résultats du travail de Filali et al [6] où cette préservation était notée dans 35% des cas. Les infirmiers et le personnel médical soignant doivent être impliqués dans la préservation du capital veineux et dans la surveillance de l’accès vasculaire [7]. Dans notre étude, les participants à l’enquête avaient les mêmes caractéristiques démographiques que ceux de l’étude de Filali et al [6]. Le patient doit aussi être bien informé sur les différentes mesures de protection du réseau veineux périphérique et central [5].

La FAV céphalique distale a été décrite par Brescia et Cimino en 1966. Elle reste le meilleur abord vasculaire [8]. La création d’une FAV nécessite une artère et une veine de bonne qualité. Le sens de circulation du sang dans l’abord vasculaire périphérique nécessite que le réseau veineux de l’anastomose artério-veineuse jusqu’au cœur, soit intègre. C’est donc le réseau veineux périphérique et le réseau veineux central qui doivent être protégés. Toute effraction de la paroi veineuse entraîne un processus de cicatrisation avec une zone de fibrose plus ou moins étendue et définitive. Le cathétérisme vasculaire (artériel ou veineux), peut s’accompagner de phlébite et/ou de thrombose [5]. Friedman et al [9] ont démontré l’importance de la consultation précoce d’un néphrologue pour la mise en place d’accès vasculaire avant l’IRCT.

La politique de préservation du réseau veineux doit être mise en place le plus précocement possible [10] chez tout patient ayant une insuffisance rénale, une maladie rénale ou une maladie générale susceptible de léser le rein (diabète, athérome, vascularites…); elle doit être poursuivie chez tout transplanté d’organe (rein, cœur, autre…). Plus généralement elle doit être appliquée chez toute personne atteinte d’une maladie chronique dont le traitement nécessite un accès répéter aux vaisseaux (mucoviscidose, hémoglobinopathies, maladies nécessitant des aphérèses, une chimiothérapie au long cours…) [5]. Aussi, l’abord vasculaire est une source de difficultés auxquelles sont confrontés quotidiennement tous les professionnels amenés à participer à la prise en charge d’un enfant [11].

Les veines des avant-bras et des bras sont les plus sollicitées pour la création d’une FAV [6]. C’est pourquoi les prélèvements sanguins et les cathétérismes veineux pour perfusions devraient être faits sur les veines du dos des mains plus nombreuses mais plus fines que les veines de l’avant bras. Les deux bras doivent être protégés de la même façon [5]. Dans notre enquête, le dos de la main était le site le plus ponctionné et le membre supérieur droit était un plus utilisé que le membre supérieur gauche (92,3% versus 81,5 %). Cela peut être expliqué par le fait que la FAV est crée de préférence au membre non dominant, c’est à dire l’avant bras gauche chez un droitier. Dans le travail de Filali et al [6]; le pli du coude était le site le plus utilisé et les deux membres supérieurs étaient ponctionnés sans distinction.

Il est impératif pour augmenter le taux de réussite d’utiliser tous les moyens disponibles pour aider au repérage veineux avant la ponction. La dilatation veineuse est un facteur de succès. Parallèlement à l’usage du garrot, le réchauffement et le passage d’alcool permettent d’augmenter le diamètre des veines [5,12]. Le garrot était le moyen le plus utilisé par nos infirmiers, seulement la moitié utilisait l’alcool et les deux tiers réchauffaient les veines. Ces habitudes peuvent être du à l’information insuffisante de nos infirmiers sur la préservation du capital vasculaire par ces moyens très simples.

Concernant le calibre des aiguilles, l’utilisation de petit calibre (20 à 24 gauge) est conseillé [5]. Dans notre travail, cette mesure n’a pas été respectée. La majorité des intranules et des épicrâniennes employées était de 18 gauge, alors que les venojects était de petit calibre dans 81% des cas.

L’incidence des complications liées au cathétérisme veineux périphérique est estimée à 20-35% des patients hospitalisés [13]. Dans notre étude, ces complications étaient notées dans 41% des cas. La lymphangite complique 20 % en moyenne des cathéters veineux périphériques alors qu’elle était plus fréquente dans notre étude. L’utilisation d’un accès temporaire par un cathéter veineux central est une source majeure de morbidité, de mortalité et de dépenses chez les malades en IRCT [14]. Leur utilisation pouvait affecter de façon négative le taux de succès des créations de FAV natives [15]. Les cathéters ont tendance à se thromboser, s’infecter et à entraîner des sténoses veineuses centrales et une dialyse inadéquate [14,16]. Le risque de développement d’une sténose apparaît élevé pour les veines sous-clavières (50 %), alors qu’il est de 10 % pour les veines jugulaires internes [17–21].

La voie sous-clavière ne devrait être utilisée que lorsque les voies jugulaires ne sont plus accessibles ou que la création d’un FAV ne puisse plus être envisagée du côté homolatéral. La voie fémorale doit être réservée aux dialyses faites en urgence et aux cathétérismes de courte durée. La voie jugulaire interne doit être la voie privilégiée pour les cathétérismes prolongés ou permanents. L’utilisation de l’écho-repérage (et éventuellement de l’échoguidage) doit être recommandée lors de la pose d’un cathéter veineux central [5].

Dans la littérature, il y a peu d’études qui évaluent le rôle de l’infirmier dans la protection du capital vasculaire. Des études ultérieures s’avèrent nécessaires afin d’assurer une meilleure préservation du réseau veineux dans cette population de patients fragiles et multi compliqués.

## Conclusion

L’information et l’éducation du personnel paramédical sont obligatoires ainsi que l’utilisation des cathéters veineux de petit calibre afin d’assurer cette protection. La politique de préservation du réseau veineux doit être poursuivie quel que soit le traitement de l’insuffisance rénale chronique: hémodialyse, dialyse péritonéale ou transplantation rénale et elle doit être maintenue quel que soit l’âge du patient.
